# A Novel Imaging Analysis Method for Capturing Pharyngeal Constriction During Swallowing

**Published:** 2016-08-25

**Authors:** Ryan W. Schwertner, Kendrea L. Garand, William G. Pearson

**Affiliations:** 1 Department of Cellular Biology & Anatomy, Medical College of Georgia, Augusta University, Augusta, GA; 2 Department of Otolaryngology-Head & Neck Surgery, Medical University of South Carolina, Charleston, SC

**Keywords:** Deglutition, Dysphagia, Biomechanics, Videofluoroscopy, Imaging analysis, Morphometrics

## Abstract

Videofluoroscopic imaging of swallowing known as the Modified Barium Study (MBS) is the standard of care for assessing swallowing difficulty. While the clinical purpose of this radiographic imaging is to primarily assess aspiration risk, valuable biomechanical data is embedded in these studies. Computational analysis of swallowing mechanics (CASM) is an established research methodology for assessing multiple interactions of swallowing mechanics based on coordinates mapping muscle function including hyolaryngeal movement, pharyngeal shortening, tongue base retraction, and extension of the head and neck, however coordinates characterizing pharyngeal constriction is undeveloped. The aim of this study was to establish a method for locating the superior and middle pharyngeal constrictors using hard landmarks as guides on MBS videofluoroscopic imaging, and to test the reliability of this new method. Twenty de-identified, normal, MBS videos were randomly selected from a database. Two raters annotated landmarks for the superior and middle pharyngeal constrictors frame-by-frame using a semi-automated MATLAB tracker tool at two time points. Intraclass correlation coefficients were used to assess test-retest reliability between two raters with an ICC = 0.99 or greater for all coordinates for the retest measurement. MorphoJ integrated software was used to perform a discriminate function analysis to visualize how all 12 coordinates interact with each other in normal swallowing. The addition of the superior and middle pharyngeal constrictor coordinates to CASM allows for a robust analysis of the multiple components of swallowing mechanics interacting with a wide range of variables in both patient specific and cohort studies derived from common use imaging data.

## Introduction

Swallowing (deglutition) is a complex sensorimotor response involving structures of the upper aerodigestive tract that results in the safe and efficient ingestion of saliva, food and liquids. Disruption of this complex event can lead to dysphagia (swallowing impairment) resulting in malnutrition, dehydration, social isolation, and even death related to aspiration pneumonia. The most commonly used instrumental examination to assess swallowing function is the modified barium swallow (MBS) study that uses real-time x-ray to permit visualization of the rapid physiologic process and allows for analysis of crucial oropharyngeal swallowing mechanics^[1]^. A key element known as the pharyngeal stripping wave is the sequential, progressive contraction of the superior, middle, and inferior pharyngeal constrictor muscles critical in assisting pharyngeal clearance of ingested material. Pressure generation in pharyngeal swallowing results from the combined action of pharyngeal constrictor muscles with muscles underlying tongue base retraction, laryngeal elevation, and pharyngeal shortening^[2]^.

Several methods have been developed to measure pharyngeal swallowing mechanics using MBS. The Modified Barium Swallow Impairment Profile™ (MBSImP^©^) was designed to capture physiologic swallowing impairment using a standardized, validated, and reliable approach for the assessment and interpretation of the MBS. The MBSImP assesses 17 physiologic components of oropharyngeal swallowing physiology, including pharyngeal stripping and contraction in the lateral and anterior-posterior viewing planes, respectively^[1]^. This approach, useful for clinical and research purposes, accounts for differences in key components of swallowing physiology that comprise an overall impression score. However, component or impression scores cannot characterize how these elements covary or interact with each other. Many displacement measurements of the hyoid, larynx, and tongue base have been described, however how univariate measurements characterize multiple overlapping components of swallowing mechanics is not addressed in these methods^[3–5]^. Pharyngeal constriction ratio uses lateral view MBS imaging to quantify pharyngeal constriction as a surrogate for pharyngeal constriction strength^[6]^. This highly useful outcome measure by itself, does not account for the complex interactions of the mechanics resulting in suboptimal pressures.

Computational analysis of swallowing mechanics (CASM) utilizes MBS imaging to analyze and visualize the covariant elements of swallowing mechanics. Currently 10 coordinates obtained from lateral view MBS imaging map hyoid movement, laryngeal elevation, pharyngeal shortening, tongue base retraction, and head and neck extension^[7,8]^. Patient specific CASM is performed by using a semi-automated MATLAB tracker tool to map coordinates in every frame of the MBS video recording at 30 frames per second^[9,10]^. Multivariate morphometric analysis of coordinates provides statistical evaluation of the shape change representing the mechanics of swallowing within a patient or cohort. It also produces a visual representation using vectors to characterize the relative contribution of each component of swallowing mechanics within a patient or cohort. Mapping the superior and middle pharyngeal constrictors would provide a more comprehensive analysis of the covariant elements of swallowing mechanics.

**Figure 1:**
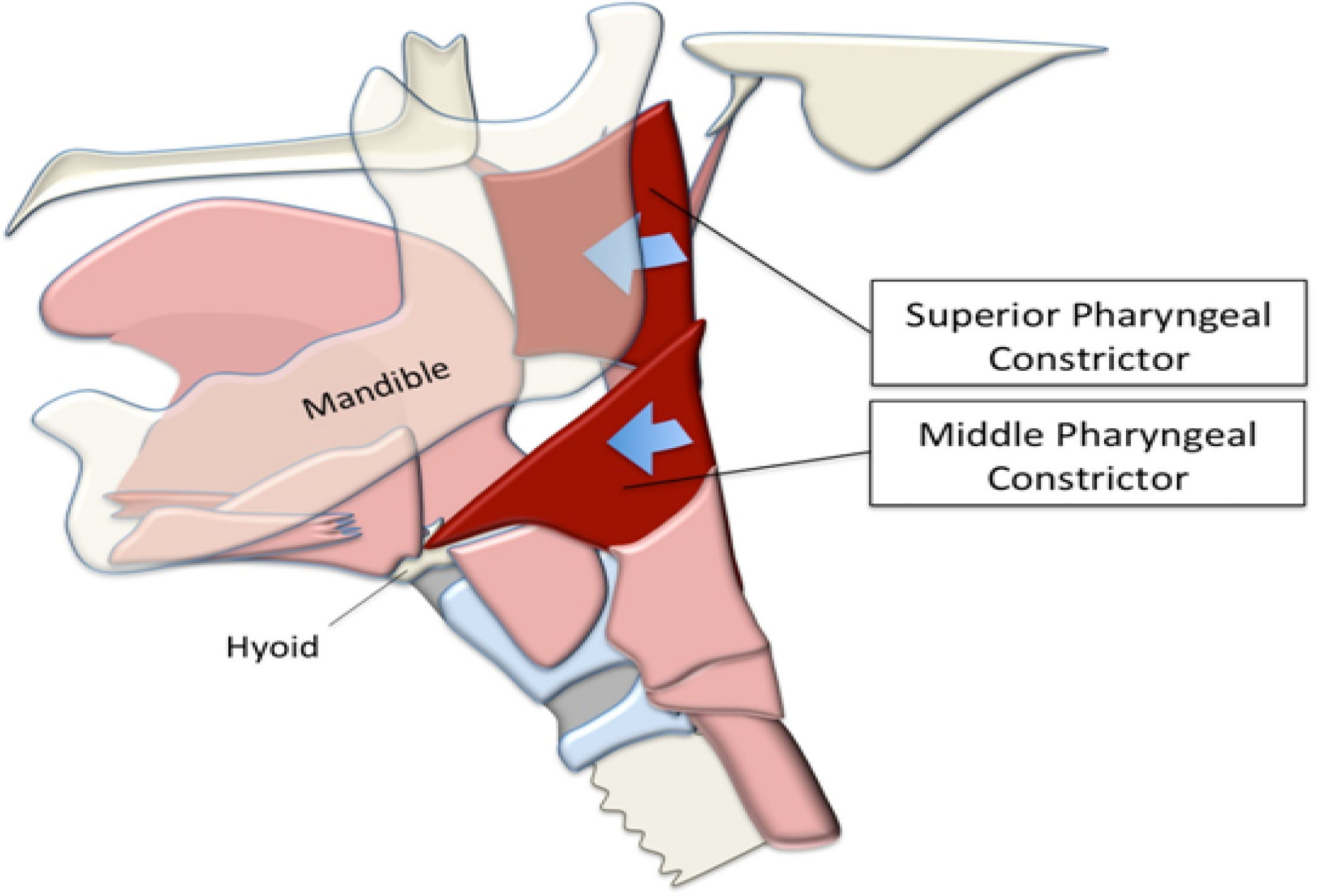
The superior pharyngeal constrictor attaches anteroinferiorly at the end of the mylohyoid line near the base of the mandible. The middle pharyngeal constrictor attaches to the greater horn of the hyoid bone.

Soft tissue anatomical landmarks, such as the pharyngeal constrictors, are difficult to map reliably^[11]^. In this study we describe our approach to annotating the superior and middle pharyngeal constrictors by using an anatomical feature that can be reliably tracked on MBS to locate the position of the muscles. The superior pharyngeal constrictor attaches anteroinferiorly at the alveolar process of the mandible and collapses toward the tongue base during swallowing. The middle pharyngeal constrictor attaches to the greater horn of the hyoid bone and contributes to the pharyngeal stripping wave^[12]^ (Figure 1). Based on these attachments, the base of the mandible and the greater horn of the hyoid were chosen to locate coordinates representing attachment sites of the superior and middle pharyngeal constrictor respectively. In the present study, we test how reliably these coordinates can be mapped in order to expand the capacity of CASM to include pharyngeal constriction. We also discuss the potential clinical benefits of this new development.

## Materials and Methods

Twenty de-identified videos were randomly selected from a database of healthy, non-dysphagic adult MBS study videos. This database is part of a large, ongoing study designed to reveal normal variation in oropharyngeal swallowing behavior that occurs as part of the aging process by incorporating a quantitative approach for measurement of physiologic observations obtained from MBS imaging approved by the Medical University of South Carolina (MUSC) Institutional Review Board for Human Research for enrollment at MUSC and the Ralph H. Johnson VA Medical Center. Participants were excluded if he/she had known history of upper aerodigestive tract surgical resections, large hiatal hernia (>2 cm), pulmonary disease, head and neck cancer, or neurologic disease.

Each eligible participant completed an MBS study according to the MBSImP protocol^[1]^. A high resolution, videofluoroscopic device (Digital Swallowing Workstation Model 7100, Kay Elemetrics Corp.) was used for signal acquisition, digital storage, and retrieval of the swallowing data. De-identified videofluoroscopic recordings were made with a resolution of 60 fields (30 frames) per second. The standardized MBSImP swallowing protocol includes a total of 12 swallow types (10 administered with the patient in the lateral viewing plane and 2 administered in the anterior-posterior viewing plane) of increasing volume and viscosity of commercially prepared barium (Varibar^®^, E-Z-EM, Inc.). For this study a 5 ml thin liquid and 5 ml pudding swallow from each subject were included. De-identified videos were analyzed at the Medical College of Georgia at Augusta University with approval from the Augusta University Institutional Review Board.

**Figure 2:**
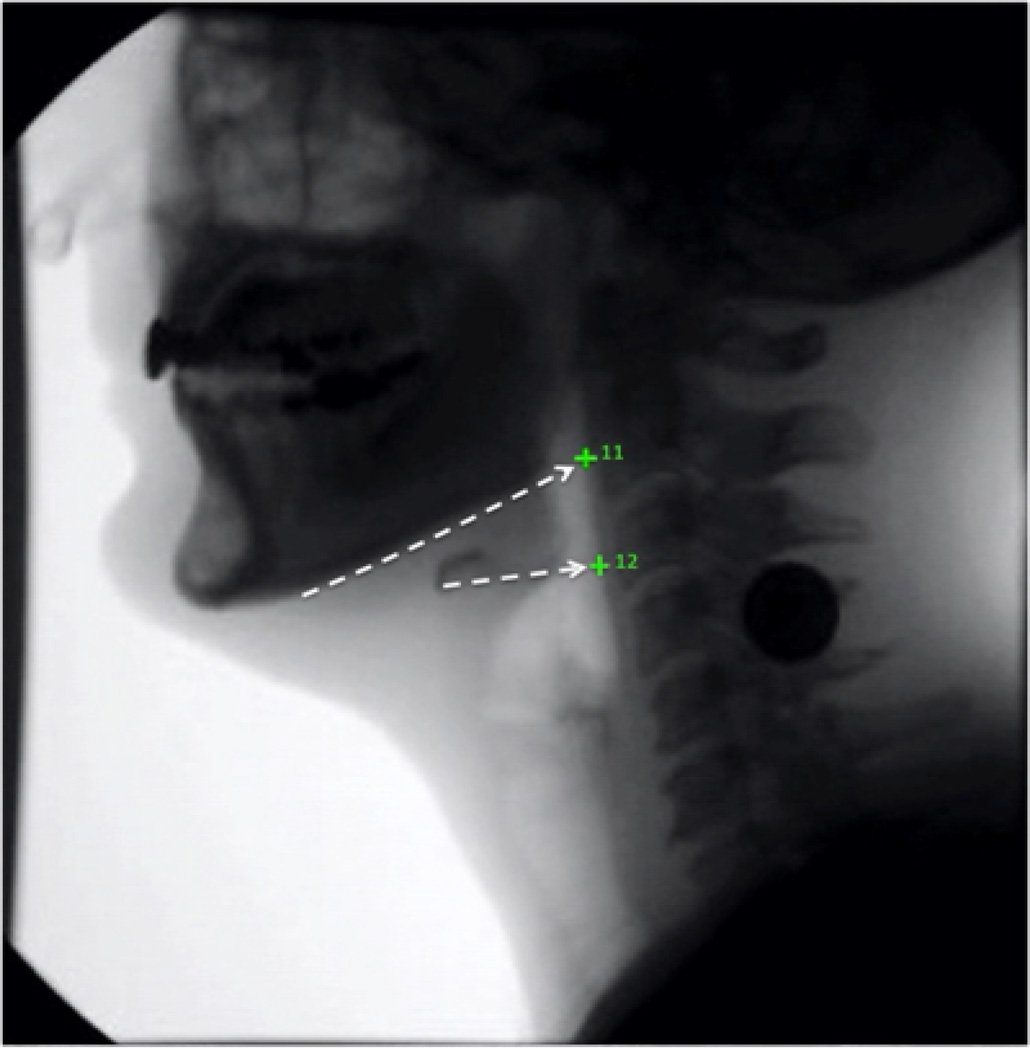
The superior pharyngeal constrictor (11) was located by following the base of the mandible posteriorly to the pharyngeal wall. The middle pharyngeal constrictor (12) was located by following the greater horn of the hyoid posteriorly to the pharyngeal wall.

After completing reliability training for the established 10 coordinates and achieving an inter-rater score r > 0.95 when compared to an expert head and neck anatomist (WP), one medical student (RS) established a protocol for annotating coordinates #11 and #12, the superior and middle pharyngeal constrictors, respectively. Coordinate #11 was tracked by visually connecting the line formed by the base of the mandible posteriorly to the pharyngeal wall. Coordinate #12 was tracked by visually connecting the line coextensive with the greater horn of the hyoid bone posteriorly to the pharyngeal wall (Figure 2). After visually inspecting five initial annotations completed by both raters, it was determined that the operational definition for coordinate #11 required adjustment. A portion of the MBS videos showed both lateral sides of the mandible due to patient rotation, which caused a superior and inferior mandibular shadow to appear on the MBS image. For these particular cases, the superior shadow was chosen as it offered a better representation of the superior pharyngeal constrictor function.

**Figure 3:**
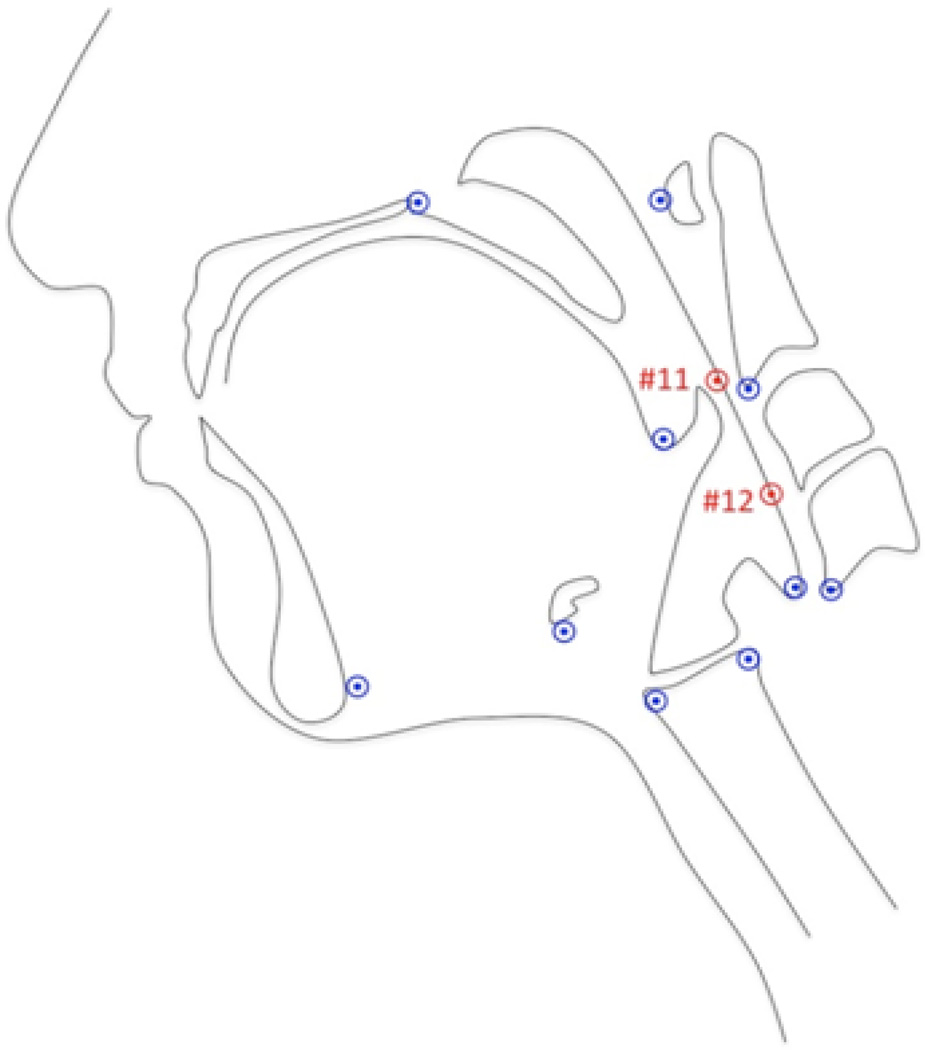
Twelve anatomical landmarks were tracked representing the skeletal base and soft tissue underlying the attachments for muscles involved in laryngeal elevation, pharyngeal shortening, and hyoid excursion during oral and pharyngeal swallowing. Points 11 and 12 approximate the superior and middle pharyngeal constrictors, respectively are pictured here in red.

A MATLAB tracker tool was used to track coordinates #11 and #12 using the new protocol in addition to the ten previously defined landmarks to test inter-rater reliability^[8,9]^ (Figure 3). Coordinates annotated from videos were exported from the MATLAB tool as text files. A Google sheet macro was used to compile the data from the individual text files into a single text file containing the data for all the videos from both raters (RS and WP). Both raters repeated annotations of the same 20 videos to determine test-retest reliability. Class 2 model Intraclass correlation coefficients (ICC) were calculated within raters and between raters at trial 1 and trial 2^[13]^. ICCs and 95% confidence intervals were determined using Excel with the Real Statistics Using Excel add-in^[14]^.

Morphometric analysis of coordinates was performed using MorphoJ integrated software^[15]^. The compiled coordinate text file was uploaded into MorphoJ. A procrustean fit to mathematically realign all coordinates was executed prior to shape analysis. A discriminate function analysis was performed using MorphoJ producing eigenvectors to visualize relative shape change of all twelve coordinates associated with swallowing. Each set of 12 coordinates was assigned a classifier variable of either oral transport or pharyngeal phase of swallowing. Oral transport was marked to begin when the bolus moved from the anterior oral cavity toward the pharynx. The frame preceding the beginning of the pharyngeal phase was marked as the end of oral transport. The first frame that demonstrated rapid anterior and superior movement of the hyoid bone was marked as pharyngeal phase onset with the end of this phase marked by closure of the upper esophageal sphincter. Eigenvectors were transposed manually in reference to the vertebral column to characterize the mechanical components of swallowing mechanics including pharyngeal constriction, hyoid movement, laryngeal elevation, tongue base retraction, pharyngeal shortening, and head and neck extension.

## Results

**Figure 4:**
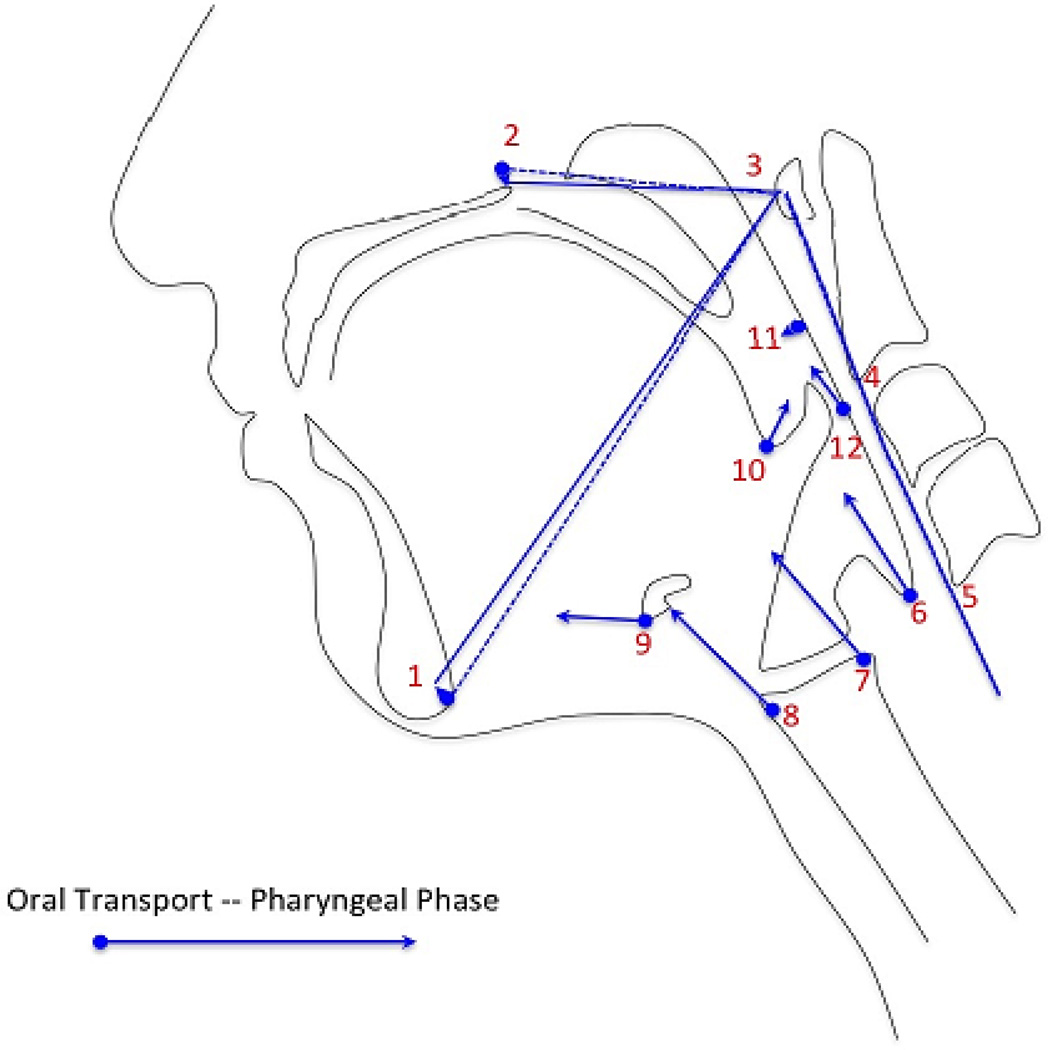
Eigenvectors characterizing the multiple covariant components of swallowing mechanics including head and neck extension (1–5), pharyngeal shortening (6),laryngeal elevation (7,8),hyoid movement (9), tongue base retraction (10), and pharyngeal constriction (11,12).

Intraclass correlation coefficients and 95% confidence intervals for each element of coordinates 11 and 12 are reported in table 1 with a range of ICC = 0.990 – 0.998 for the test-retest inter-rater reliability (Rater 1 vs. Rater 2 at Trial 2). Eigenvectors characterizing swallowing in the subject cohort are reported in figure 4.

## Discussion

This study found that coordinates mapping the function of the superior and middle pharyngeal constrictors are reliably mapped utilizing hard landmarks as guides to locate muscle position in lateral view MBS imaging. Adding these two coordinates to CASM provides an unprecedented opportunity to evaluate multiple elements of swallowing mechanics as they interact with variables of interest in swallowing research, such as patient demographic and clinical characteristics, medical comorbidities, effects of medical/surgical and behavioral treatment, oral intake status, and bolus properties.

Intra-rater reliability was higher for rater 1 than rater 2, though inter-reliability at trial 2 was excellent (ICC range = 0.99–1.00). We believe this was due to the training effect since rater 1 developed the method. It has been argued that Pearson correlations are sufficient for test-retest reliability in these cases since error is reduced with training^[16]^. However, here, the more rigorous analysis was left in place. These data suggest that new raters can be effectively trained and achieve superior reliability over time. A key limitation is that rater 2 is an expert head and neck anatomist. The amount of time required to train a novice learner is an open question.

One common source of variance in imaging analysis of swallowing mechanics using MBS imaging includes movement of the patient during swallowing, especially the flexion and extension of the head and neck^[17]^. CASM employs shape change analysis to characterize swallowing mechanics. This analysis begins with a Procrustes superimposition, a mathematical realignment of all coordinates that scales, translates and rotates the constellation of coordinates for statistical analysis to control for image rotation, patient movement, and size differences.

Radial distortion and magnification of the image can also be a factor in imaging analysis where muscle function is measured by displacement measurements using an international system of units such as millimeters. In shape change analysis, this variance is mitigated as coordinates are analyzed in morphospace. Morphometric analysis is concerned with the scatter plot variance of each coordinate in context of the overall shape rather than the actual distance between coordinates.

One limitation of CASM is that eigenvectors, which characterize the variance of shape defined by coordinate position associated with some named independent variable of interest, are not the same as a kinematic measurement. The analysis does not provide information, for example, of how far the hyoid moves in one swallow when compared with another in any standard form of measurements. However, the analysis does provide information about how the various component elements of swallowing mechanics interact with each other associated with any named variable of interest. With the addition of coordinates #11 and #12, evaluating the function of the pharyngeal constrictor muscles in relationship with muscles that retract the tongue base, elevate the larynx, and shorten the pharynx is now possible.

Vectors characterizing the interaction between swallowing elements reported in Figure 4 represent normal swallowing as previously described in the literature^[18]^. The collapsing of the pharynx to generate pressure to propel a bolus through the hypopharynx involves not only the pharyngeal constrictor muscles, but also the contribution of the long pharyngeal muscles that shorten the pharynx and elevate the larynx as well as muscles that retract the tongue base^[19]^. Future work will use this method of image analysis to investigate unanswered questions about swallowing function and dysfunction.

We have demonstrated that coordinates representing the function of the superior and middle pharyngeal constrictor can be reliably included in the CASM method allowing for a more robust study of mechanics in human swallowing using imaging.

## Figures and Tables

**Table 1:** 

	11x	11y	12x	12y
**Trial 1 (Rater 1 vs. Rater 2) ICC**	0.915	0.935	0.919	0.928
**lower**	0.749	0.683	0.741	0.709
**upper**	0.959	0.974	0.962	0.970
**Trial 2 (Rater 1 vs. Rater 2) ICC**	0.990	0.998	0.992	0.998
**lower**	0.989	0.998	0.990	0.998
**upper**	0.991	0.999	0.993	0.999
**Rater 1 (Trial 1 vs. Trial 2) ICC**	1.000	1.000	1.000	1.000
**lower**	1.000	1.000	1.000	1.000
**upper**	1.000	1.000	1.000	1.000
**Rater 2 (Trial 1 vs. Trial 2) ICC**	0.906	0.926	0.910	0.927
**lower**	0.742	0.650	0.754	0.653
**upper**	0.953	0.971	0.955	0.971
